# iHMS: a database integrating human histone modification data across developmental stages and tissues

**DOI:** 10.1186/s12859-017-1461-y

**Published:** 2017-02-11

**Authors:** Yanglan Gan, Han Tao, Jihong Guan, Shuigeng Zhou

**Affiliations:** 1 School of Computer Science and Technology, Donghua University, Shanghai, China; 2 Department of Computer Science and Technology, Tongji University, Shanghai, China; 3 Shanghai Key Lab of Intelligent Information Processing and School of Computer Science, Fudan University, Shanghai, China

**Keywords:** Human histone modification, Developmental stages, Data integration, Database

## Abstract

**Background:**

Differences in chromatin states are critical to the multiplicity of cell states. Recently genome-wide histone modification maps of diverse human developmental stages and tissues have been charted.

**Description:**

To facilitate the investigation of epigenetic dynamics and regulatory mechanisms in cellular differentiation processes, we developed iHMS, an integrated human histone modification database that incorporates massive histone modification maps spanning different developmental stages, lineages and tissues (http://www.tongjidmb.com/human/index.html). It also includes genome-wide expression data of different conditions, reference gene annotations, GC content and CpG island information. By providing an intuitive and user-friendly query interface, iHMS enables comprehensive query and comparative analysis based on gene names, genomic region locations, histone modification marks and cell types. Moreover, it offers an efficient browser that allows users to visualize and compare multiple genome-wide histone modification maps and related expression profiles across different developmental stages and tissues.

**Conclusion:**

iHMS is of great helpfulness to understand how global histone modification state transitions impact cellular phenotypes across different developmental stages and tissues in the human genome. This extensive catalog of histone modification states thus presents an important resource for epigenetic and developmental studies.

## Background

Nearly all cells of an organism share the same genome but exhibit diverse phenotypes and carry out dramatically different functions. In eukaryotic cells the genome is organized into chromatin. Cell-type specific chromatin organization enables differential access and activity of regulatory elements and the manifestation of unique cellular phenotypes [1, 2]. Recent genome-wide studies have shown that cooperative chromatin modifications affect the structure of chromatin, shape the macro-environment of DNA, and add an extra layer of information to the genome sequence [3, 4]. These chromatin states are distinctive for different developmental stages [5], tissues [6], and disease states [7, 8], which can play important roles in establishing cell identity during development [9]. Therefore, studying histone modification states of multiple developmental stages and cell types may extend the knowledge of epigenetic dynamics and regulatory mechanisms in cellular differentiation, reprogramming, and disease processes.

Large-scale mapping of histone modifications has emerged as a powerful means for characterizing chromatin structures. The technology of chromatin immunoprecipitation followed by sequencing (Chip-Seq) can interrogate chromatin structure across the genome [10], which is increasingly applied for charting genome-wide maps of histone modifications [11, 12]. Currently, a large collection of histone modification maps are being generated for diverse developmental stages, lineages and tissues, with the emphasis on mammalian models [5, 13–17]. The expanding body of epigenomic data provides an opportunity to elucidate novel relationships among various histone modifications [18, 19], to characterize regulatory elements in the human genome [20], and to understand how global features of histone modifications impact cellular phenotypes across different developmental stages, lineages, and environmental conditions [21, 22].

Bearing these promises, a fundamental problem is to integrate histone modification maps of diverse developmental stages and tissues in the public domain. Over the past years, a few epigenomic databases have been developed for the integration of various human epigenomic data from different tissues and experiments [23–25]. These widely used databases are designed to catalyze basic biology and disease-oriented research, and mainly provide researchers with a resource for visualizing and downloading whole-genome datasets. However, it is not intuitive and easy to conduct detailed queries and comparisons for specific histone modification states of interested genomic regions. Another human histone modification database HHMD [26] includes only epigenomic data of several cell types, rather than integrate histone modification data from multiple developmental stages. Obviously, there is an urgent need to construct a specialized database that comprehensively provides high-resolution genome-wide histone modification data for epigenetic and developmental studies.

Here, we report a database iHMS that integrates human histone modification data covering diverse developmental stages and primary tissues. It also includes genome-wide expression data of different conditions and reference genes, GC content and CpG island information. iHMS has an intuitive and user-friendly query interface, which enables both basic and advanced search based on gene names/genomic region locations, histone modification marks and cell types, as three major query options. Moreover, it allows users to visualize and compare multiple genome-wide histone modification maps and related expression profiles at different developmental stages and tissues via a powerful browser. Thus, iHMS can provide a systematic view of the dynamic histone modification landscapes during cellular differentiation and development, which is useful for researchers to compare the variability of histone modification states with underlying gene expression, to identify cell-type-specific histone modification states and their regulatory implications for cellular phenotypes across different developmental stages and tissues.

## Construction and content

### Database overview

**Fig. 1:**
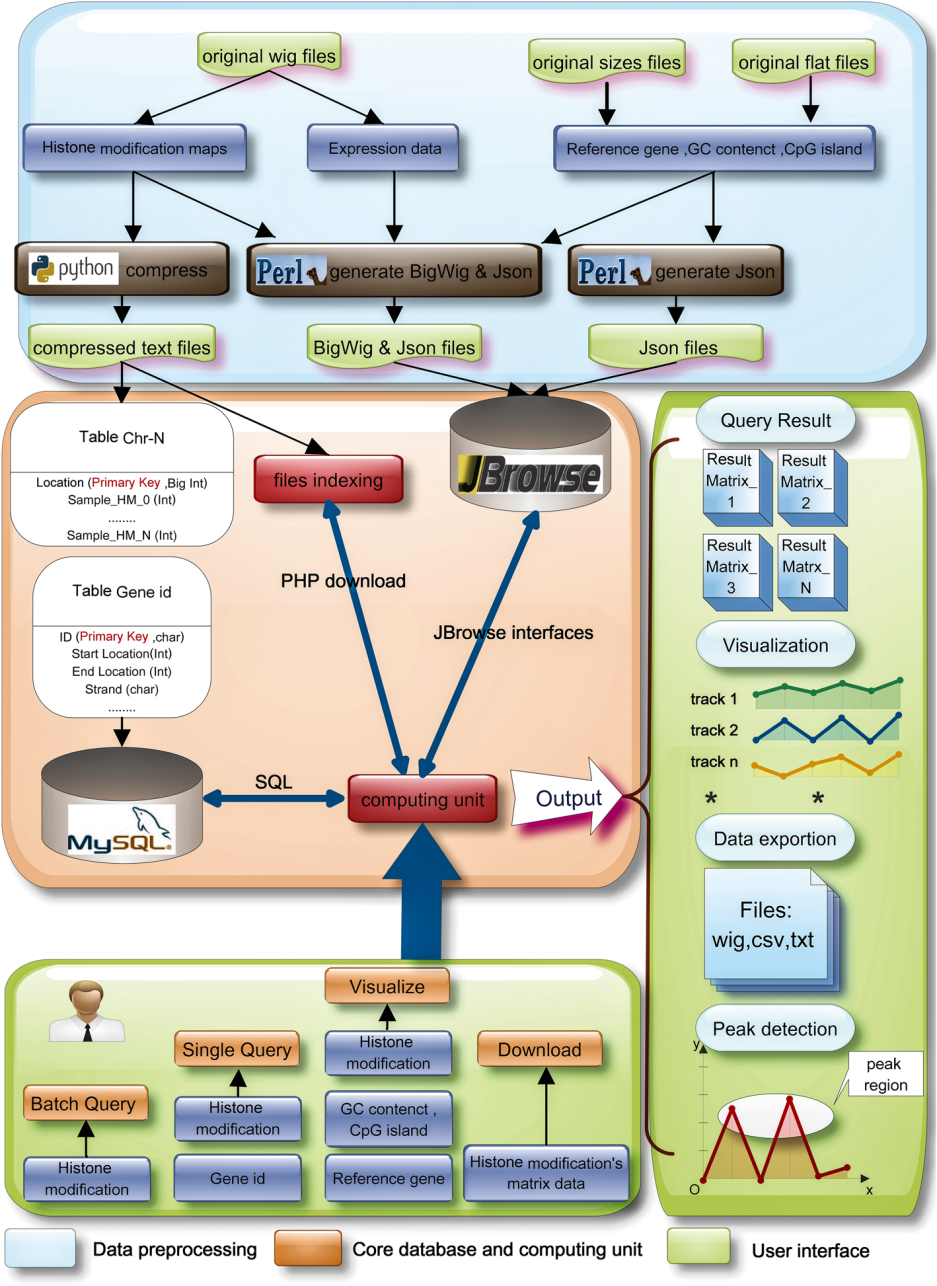
An overview of the iHMS database. iHMS integrates genome-wide histone modification maps of different developmental stages and tissues of human. Related gene expression profiles and genomic annotations are also incorporated. iHMS is composed of *three layers*: data preprocessing, core database and computing unit, and user interface

In human, the study of epigenetic mechanisms underlying the regulation of early embryonic development requires access to large amounts of epigenomic data in different developmental stages. iHMS is a web-based integrated platform that enables users to query, compare, analyze and visualize genome-wide histone modification patterns across different human developmental stages and representative tissues. Figure 1 shows the framework of iHMS, which is composed of three layers: data preprocessing, core database and computing unit, and user interface.

In the data preprocessing layer, we reorganize and compress the collected raw data. This process is of great importance to reduce the redundancy among different datasets, to improve query efficiency, and to facilitate data usage in the follow-up analysis. In the second layer, the compressed histone modification maps are imported and stored into a relational database. All histone modification maps are stored in tables to enable efficient management, search and representation. The related expression data and genomic annotations, including reference genes, GC content and CpG island information, are stored in file system for quick access by the JBrowse visualization system [27]. To bridge the core database and the user interface, a computing unit is also developed in the second layer. It processes the dataset according to users’ requests from the user interface to guarantee prompt responses. The user interface layer of iHMS provides users with friendly and interactive interfaces for data query, visualization, download and peak detection analysis. With these interfaces, users can easily configure options to access data from the core database. Results of search and analysis will be presented to users and obtained in files. The web-based browser is built for manipulating and displaying these datasets on the whole genome.

### System implementation

The integrated system iHMS is built with LampServer (Linux+Apache+MySQL +PHP). LampServer is a fast and open source development environment, allowing users to develop web applications with Apache, PHP and MySQL. Raw datasets of histone modification maps are reorganized and compressed by Perl and Python scripts. The core database of iHMS is implemented with MySQL relational database system (version 5.6.12). To facilitate efficient management and query, all histone modification maps are stored in MySQL tables, whereas the expression data and genomic annotations are stored in files for quick access by JBrowse. The computing unit is implemented by PHP, a server-side scripting language designed for web development. The browser-based interfaces are developed with a collection of web development techniques, including JavaScript, CSS and Ajax. These powerful techniques make data access simple and efficient. To display histone modification profiles of specified genomic regions, we apply Highcharts.js and CanvasJS, which are both effective and open-source painting galleries. Specifically, the Ajax technique enables data to be transferred between server and browser asynchronously without interfering with the display of the current web page. Meanwhile, we implement and integrate a peak calling method in the interface, allowing users to identify enrichment sites of different histone modification marks. On the basis of the processed bin-based data, the peak calling procedure first determines a significant enrichement threshold by a percentile rank statistic method and utilizes Monte Carlo simulation method to control false discovery rate [28]. For the visualization of genome-wide histone modification maps, the interactive and user-friendly browser is built on JBrowse, which requires light resource and facilitates fast scrolling and zooming on the whole genome.

### Data collection and preprocessing

**Fig. 2:**
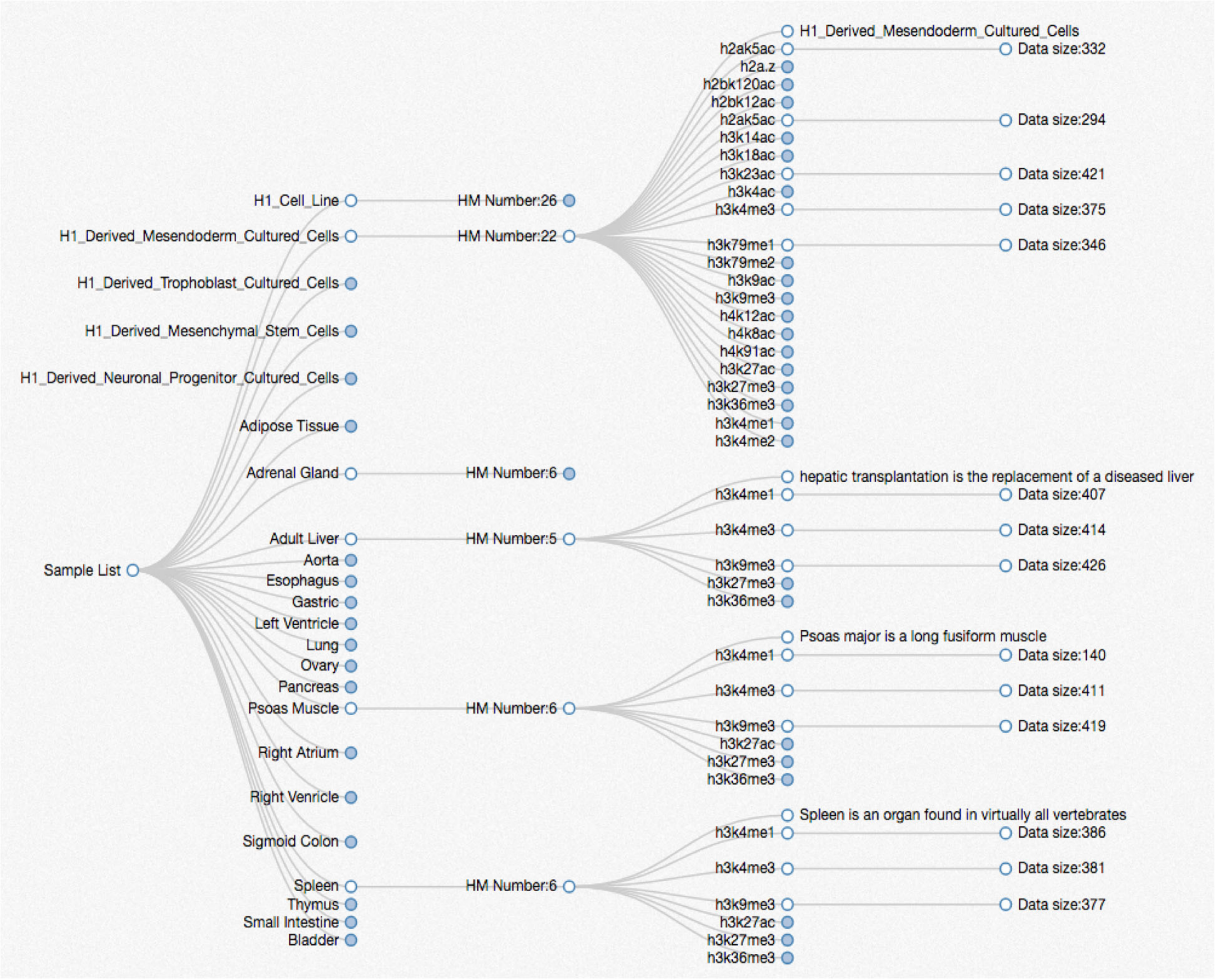
The summary of iHMS data content. More detailed information can be viewed on the web page by clicking each *solid* node

iHMS integrates a collection of over 200 histone modification maps for phenotypically diverse human developmental stages and tissues, produced by the NIH Roadmap Epigenomics Mapping Consortium [5, 16]. These epigenetic maps depict the dynamic landscapes of important histone modifications. In recent studies, to investigate early human developmental decisions, H1 human embryonic stem cells (hESCs) were differentiated into a variety of precursor cell types [5, 14], including trophoblast-like cells (TBL) [29], mesendoderm (ME) [30], neural progenitor cells (NPCs) [31], and mesenchymal stem cells (MSCs) [32]. These lineages represent extra-embryonic and embryonic lineages at early stages of development. Specifically, the first three states represent developmental events that mirror critical developmental decisions in the embryo. MSCs are fibroblastoid cells that are capable of expansion and multi-lineage differentiation to bone, cartilage, adipose, muscle, and connective tissues [28]. Also, H9 human embryonic stem cells were differentiated into neurons and neural progenitors [33]. Accordingly, the subsequent primary tissues, representatives of all three germlayers were also investigated, including adipose, adrenal gland, adult liver, aorta, esophagus, gastric, left ventricle, lung, ovary, pancreas, psoas muscle, right ventricle, right atrium, sigmoid colon, spleen, thymus, small intestine, breast, brain and bladder. In these tissues, genome-wide maps of major chromatin marks were generated using ChIP-seq [5]. In detail, the chromatin marks including H3K4me1/2/3, H3K36me3, H3K9me3, H3K27me3, H3K79me1, H2AK5ac, H2bK120ac, H2BK5ac, H3K18ac, H3K23ac, H3K27ac, H3K4ac, H3K9ac and H4K8ac were profiled. As gene expression correlates closely with histone modification status, expression profiles of the investigated cell types were also incorporated in iHMS. RNA expression profiles of these cell types were generated by RNA-seq technology. The summary of these datasets is shown in Fig. 2. On the webpage, we can view more detailed information by clicking each solid node.

**Fig. 3:**
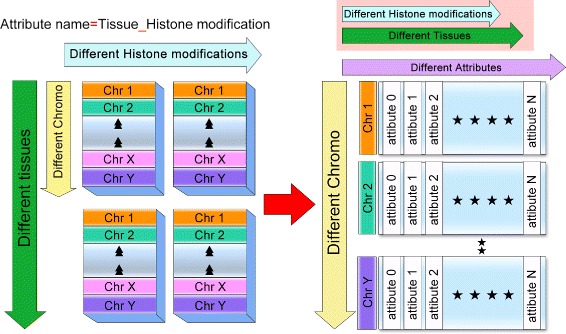
The illustration of data redundancy reduction. Histone modification datasets are reorganized and compressed before being imported to the core database

To efficiently integrate and access these datasets, we preprocessed the raw data in the following steps. The whole-genome data were divided into non-overlapping 200 bp bins. Then, we summed neighboring reads and assigned an integer for each bin. We obtained a total 288 genome-wide data sets, including 254 histone modification data sets, 34 RNA-seq data sets, covering 8 early developmental cell lines and 26 primary tissues, representatives of all three germlayers. These integrated datasets enable in-depth investigation of histone modification data, and facilitate users to explore the dynamic histone modifications and transcriptional changes that drive developmental fate decisions. These histone modification datasets were originally classified by cell type and histone modification mark. In this way, the genomic locations were repeatedly recorded, leading to severe data redundancy. Thus, to reduce the redundancy and save the storage cost, we further reorganized and compressed these processed data sets, as shown in Fig. 3. The histone modification data of different tissues were reorganized into matrices. In the matrix, the rows represent different genomic locations, the columns indicate different attributes. Here, each attribute is a combination of the tissue name and histone modification mark (eg H1_h3k4me3). The processed datasets became simpler and more compact for the following indexing and analysis. The scripts of these preprocessing steps and the processed data were deposited into the repository Figshare, which can be downloaded from the following link (https://figshare.com/s/f52b6032884637984711).

## Utility and discussion

### Data query

**Fig. 4:**
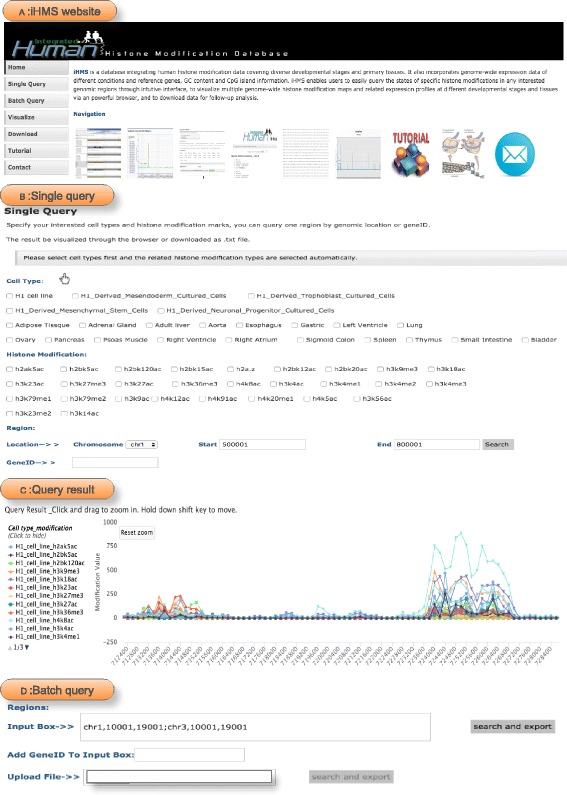
Screenshots of data query in iHMS. This figure describes the search for multiple histone modification marks of a specific cell type. **a** In the Entry Screen, we choose *Single* Query. **b** A search for all profiled histone modification marks of H1 *cell line*. **c** The matched results are displayed on the *bottom* of the query page. Each line in the graph represents the histone modification states along the genomic region. By clicking the line on the *left panel*, we can view or hide the corresponding histone modification mark. **d** In the Batch Query page, multiple genomic locations can be searched at the same time

The iHMS database can be accessed through web interface to search for specific histone modification states of any interested regions across different developmental stages and tissues (Fig. 4 a). The single query page allows searching interested region by genomic location or gene ID. For users’ convenience, the matched genes will be recommended to select when users input part of the geneId, and the gene locations are auto-completed in the location input fields. In support of comparative and differential analysis, iHMS offers users with different options: (i) query by developmental stages/tissues, and (ii) query by histone modification marks, as shown in Fig. 4 b. By selecting a specific development stage or tissue and multiple histone modification marks, users can compare the histone modification patterns and identify combinatorial modification patterns among theses marks. In the second way, users are able to search for a particular histone modification mark in multiple developmental stages or tissues, which facilitates the differential analysis. Furthermore, users are allowed to submit more comprehensive queries by combining multiple histone modification marks and cell types. Once the search is finished, all the matched results are displayed at the bottom of the webpage (Fig. 4 c). By clicking the label on the left panel, the profile of each histone modification could be viewed or hided. To check the details of the matched results, users can zoom in and move to any interested local region in the figure. All results can be saved in csv format for downstream analysis.

We also designed a batch query option for users, which allows searching for many interested genomic locations at the same time. To conduct batch query, the developmental stages/tissues and the histone modification marks are set as in the single query page. The locations of multiple regions or the interested gene list can be uploaded from files or submitted in the input box (Fig. 4 d). After clicking the search and export button, users can retrieve the matched results in a txt file [34].

### Visualization and comparison

**Fig. 5:**
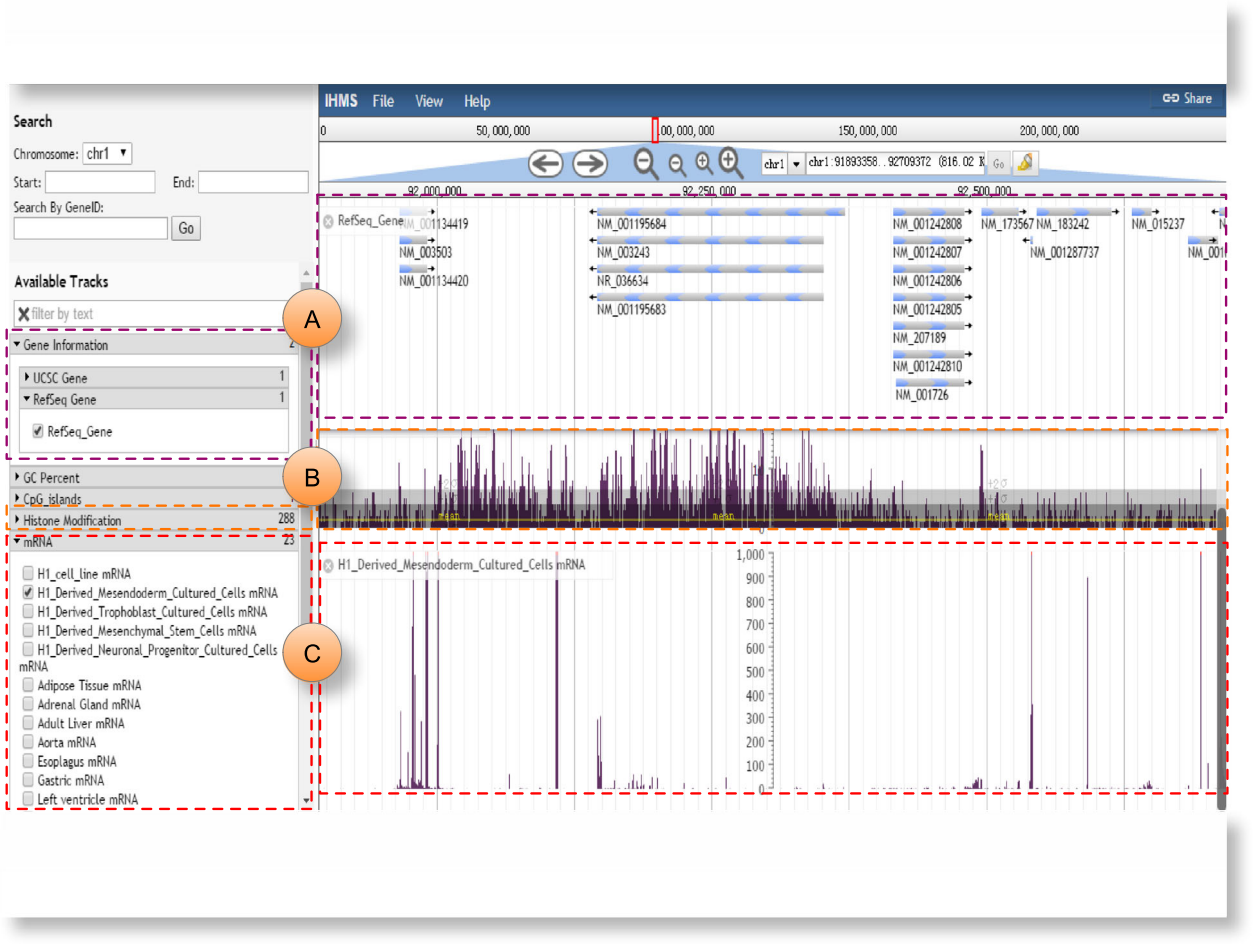
Snapshots of visualization results in iHMS. **a** Gene information, GC content and CpG island. **b** Histone modification profiles of interested developmental stages and tissues. **c** Gene expression profiles

To visualize histone modification maps on a genome-wide scale, iHMS deploys a user-friendly and interactive browser which is built on JBrowse. It provides an integrated visualization tool for viewing different histone modification marks, gene expression, reference gene annotations, GC content and CpG island information (Fig. 5). For all available developmental stages or tissues, users could easily specify interested tracks to display by clicking the icons on the left panel. Also, users are able to browse, zoom and scroll any region along the genome. By clicking a gene or region on a specific track, the corresponding details will be displayed. For example, on clicking a gene on the RefSeq gene profile, the related annotations will pop up.

With the browser, users are able to conduct further analysis. On one hand, the browser is of usefulness to compare the histone modification states of specific genes or regions at different developmental stages by taking account of multiple relevant data tracks. It facilitates users to identify tissue-restricted histone modification patterns, which is important in maintaining the identity of cell/tissue type. On the other hand, as iHMS enable users to visualize high-resolution gene expression data as well as histone modification profiles in an interactive manner, it can also help identify the correlation between histone modification pattern and gene expression level, and develop new hypotheses regarding the regulatory functions of these chromatin features in the cell differentiation process.

### Download

Considering that researchers may need these datasets for downstream analysis, iHMS provides an effective interface for data download. The whole-genome histone modification maps and gene expression data can be downloaded in the txt format. iHMS allows users to download data by chromosome. Selecting one chromosome, users can download all available histone modification maps and gene expression data at different developmental stages and tissues. The downloaded data is processed and reorganized as matrix. Rows represents the genomic locations, while columns are the histone modification marks of different cell types. Also, genome-wide expression data across all developmental stages and tissues can be downloaded.

## Conclusions

The expanding body of chromatin data in public domain has fostered many computational efforts that aim to integrate different data types. Different from previous databases, iHMS focuses on (1) integrating the whole genome histone modification maps covering a wide spectrum of developmental states, including embryonic stem cells, early embryonic lineages and somatic primary tissue types; (2) relating histone modification maps with other related omics data, including gene expression data and sequence-based genome annotations, which allows the investigation between histone modification and gene expression; (3) enabling detailed query and comparison of histone modification states of different developmental stages and tissues for specific genomic regions or genes. The query result can be retrieved as matrix, which is convenient for further comparison and analysis; and (4) building an efficient browser for visualization of all types of data in a genome-wide manner. In general, through integration of histone modification maps with expression profiles and sequence-based genome annotations, iHMS enables the discovery of cell-type specific functional histone modification states, and the gaining of insights into the epigenetic basis of cellular phenotypes across different developmental stages and tissues. This extensive catalog of histone modification states thus provides wealth information of chromatin structure and function, which may help researchers understand the epigenetic mechanisms of the differentiation and development processes.

In the foreseeable future, more histone modification data will become available with the rapid advancement of high-throughput sequencing technologies. Further development for iHMS will integrate whole-genome histone modification data from more developmental stages and tissues of human. Meanwhile, iHMS will continually incorporate the gene expression data and interconnect them with the histone modification data. As the amount of histone modification data increases, it is also important to develop efficient web tools to support quick incorporation and analysis of these newly produced data.

## Availability and requirements

The iHMS is freely available online at http://www.tongjidmb.com/human/index.html.

## Abbreviations

Chip-Seq: chromatin immunoprecipitation followed by sequencing
hESCs: H1 human embryonic stem cells
ME: mesendoderm
MSCs: mesenchymal stem cells
NPCs: neural progenitor cells
TBL: trophoblast-like cells

## References

[1] Rivera CM, Ren B (2013). Mapping human epigenomes. Cell.

[2] Zhou VW, Goren A, Bernstein BE (2011). Charting histone modifications and the functional organization of mammalian genomes. Nat Rev Genet.

[3] Kouzarides T (2007). Chromatin modifications and their function. Cell.

[4] Bonasio R, Tu S, Reinberg D (2010). Molecular signals of epigenetic states. Science.

[5] Xie W, Schultz MD, Lister R, Hou Z, Rajagopal N, Ray P, Whitaker JW, Tian S, Hawkins RD, Leung D (2013). Epigenomic analysis of multilineage differentiation of human embryonic stem cells. Cell.

[6] Bonn S, Zinzen RP, Girardot C, Gustafson EH, Perez-Gonzalez A, Delhomme N, Ghavi-Helm Y, Wilczyński B, Riddell A, Furlong EE (2012). Tissue-specific analysis of chromatin state identifies temporal signatures of enhancer activity during embryonic development. Nat Genet.

[7] Feinberg AP (2007). Phenotypic plasticity and the epigenetics of human disease. Nature.

[8] Murphy PJ, Cipriany BR, Wallin CB, Ju CY, Szeto K, Hagarman JA, Benitez JJ, Craighead HG, Soloway PD (2013). Single-molecule analysis of combinatorial epigenomic states in normal and tumor cells. Proc Natl Acad Sci.

[9] Vastenhouw NL, Schier AF (2012). Bivalent histone modifications in early embryogenesis. Curr Opin Cell Biol.

[10] Park PJ (2009). Chip–seq: advantages and challenges of a maturing technology. Nat Rev Genet.

[11] Furey TS (2012). Chip–seq and beyond: new and improved methodologies to detect and characterize protein–dna interactions. Nat Rev Genet.

[12] O’Geen H, Echipare L, Farnham PJ (2011). Using chip-seq technology to generate high-resolution profiles of histone modifications. Methods Mol Biol.

[13] Ernst J, Kheradpour P, Mikkelsen TS, Shoresh N, Ward LD, Epstein CB, Zhang X, Wang L, Issner R, Coyne M (2011). Mapping and analysis of chromatin state dynamics in nine human cell types. Nature.

[14] Gifford CA, Ziller MJ, Gu H, Trapnell C, Donaghey J, Tsankov A, Shalek AK, Kelley DR, Shishkin AA, Issner R (2013). Transcriptional and epigenetic dynamics during specification of human embryonic stem cells. Cell.

[15] Barski A, Cuddapah S, Cui K, Roh TY, Schones DE, Wang Z, Wei G, Chepelev I, Zhao K (2007). High-resolution profiling of histone methylations in the human genome. Cell.

[16] Bernstein BE, Stamatoyannopoulos JA, Costello JF, Ren B, Milosavljevic A, Meissner A, Kellis M, Marra MA, Beaudet AL, Ecker JR (2010). The nih roadmap epigenomics mapping consortium. Nat Biotechnol.

[17] Mikkelsen TS, Ku M, Jaffe DB, Issac B, Lieberman E, Giannoukos G, Alvarez P, Brockman W, Kim TK, Koche RP (2007). Genome-wide maps of chromatin state in pluripotent and lineage-committed cells. Nature.

[18] Yu P, Xiao S, Xin X (2013). Spatiotemporal clustering of the epigenome reveals rules of dynamic gene regulation. Genome Res.

[19] Ernst J, Kellis M (2012). Chromhmm: automating chromatin-state discovery and characterization. Nat Methods.

[20] Bogdanović O, Fernandez-Miñán A, Tena JJ, de la Calle-Mustienes E, Hidalgo C, van Kruysbergen I, van Heeringen SJ, Veenstra GJC, Gómez-Skarmeta JL (2012). Dynamics of enhancer chromatin signatures mark the transition from pluripotency to cell specification during embryogenesis. Genome Res.

[21] Zhu J, Adli M, Zou JY, Verstappen G, Coyne M, Zhang X, Durham T, Miri M, Deshpande V, De Jager PL (2013). Genome-wide chromatin state transitions associated with developmental and environmental cues. Cell.

[22] Sheffield NC, Thurman RE, Song L, Safi A, Stamatoyannopoulos JA, Lenhard B, Crawford GE, Furey TS (2013). Patterns of regulatory activity across diverse human cell types predict tissue identity, transcription factor binding, and long-range interactions. Genome Res.

[23] Fingerman IM, McDaniel L, Zhang X, Ratzat W, Hassan T, Jiang Z, Cohen RF, Schuler GD (2011). Ncbi epigenomics: a new public resource for exploring epigenomic data sets. Nucleic Acids Res.

[24] Zhou X, Maricque B, Xie M, Li D, Sundaram V, Martin EA, Koebbe BC, Nielsen C, Hirst M, Farnham P (2011). The human epigenome browser at washington university. Nat Methods.

[25] IHEC data portal. http://epigenomesportal.ca/ihec/index.html. Accessed 2 June 2016.

[26] Zhang Y, Lv J, Liu H, Zhu J, Su J, Wu Q, Qi Y, Wang F, Li X (2010). Hhmd: the human histone modification database. Nucleic Acids Res.

[27] Skinner ME, Uzilov AV, Stein LD, Mungall CJ, Holmes IH (2009). Jbrowse: a next-generation genome browser. Genome Res.

[28] Lan X, Bonneville R, Apostolos J, Wu W, Jin VX (2011). W-chipeaks: a comprehensive web application tool for processing chip-chip and chip-seq data. Bioinformatics.

[29] Xu RH, Chen X, Li DS, Li R, Addicks GC, Glennon C, Zwaka TP, Thomson JA (2002). Bmp4 initiates human embryonic stem cell differentiation to trophoblast. Nat Biotechnol.

[30] Yu P, Pan G, Yu J, Thomson JA (2011). Fgf2 sustains nanog and switches the outcome of bmp4-induced human embryonic stem cell differentiation. Cell Stem Cell.

[31] Chen G, Gulbranson DR, Hou Z, Bolin JM, Ruotti V, Probasco MD, Smuga-Otto K, Howden SE, Diol NR, Propson NE (2011). Chemically defined conditions for human ipsc derivation and culture. Nat Methods.

[32] Vodyanik MA, Yu J, Zhang X, Tian S, Stewart R, Thomson JA, Slukvin II (2010). A mesoderm-derived precursor for mesenchymal stem and endothelial cells. Cell Stem Cell.

[33] Zhu J, Adli M, Zou JY, Verstappen G, Coyne M, Zhang X, Durham T, Miri M, Deshpande V, De Jager PL (2013). Genome-wide chromatin state transitions associated with developmental and environmental cues. Cell.

[34] Kent WJ, Zweig AS, Barber G, Hinrichs AS, Karolchik D (2010). Bigwig and bigbed: enabling browsing of large distributed datasets. Bioinformatics.

